# Suppressive Role of Bam32/DAPP1 in Chemokine-Induced Neutrophil Recruitment

**DOI:** 10.3390/ijms22041825

**Published:** 2021-02-12

**Authors:** Li Hao, Aaron J. Marshall, Lixin Liu

**Affiliations:** 1 Department of Anatomy, Physiology and Pharmacology, College of Medicine, University of Saskatchewan, Saskatoon, SK S7N5E5, Canada; lih887@usask.ca; 2 Department of Immunology, University of Manitoba, Winnipeg, MB R3E0T5, Canada; Aaron.Marshall@umanitoba.ca

**Keywords:** Bam32, neutrophil recruitment, CXCL2, small GTPases

## Abstract

Bam32 (B cell adaptor molecule of 32 kDa) functions in the immune responses of various leukocytes. However, the role of neutrophil Bam32 in inflammation is entirely unknown. Here, we determined the role of Bam32 in chemokine CXCL2-induced neutrophil chemotaxis in three mouse models of neutrophil recruitment. By using intravital microscopy in the mouse cremaster muscle, we found that transmigrated neutrophil number, neutrophil chemotaxis velocity, and total neutrophil chemotaxis distance were increased in Bam32^−/−^ mice when compared with wild-type (WT) mice. In CXCL2-induced mouse peritonitis, the total emigrated neutrophils were increased in Bam32^−/−^ mice at 2 but not 4 h. The CXCL2-induced chemotaxis distance and migration velocity of isolated Bam32^−/−^ neutrophils in vitro were increased. We examined the activation of small GTPases Rac1, Rac2, and Rap1; the levels of phospho-Akt2 and total Akt2; and their crosstalk with Bam32 in neutrophils. The deficiency of Bam32 suppressed Rap1 activation without changing the activation of Rac1 and Rac2. The pharmacological inhibition of Rap1 by geranylgeranyltransferase I inhibitor (GGTI298) increased WT neutrophil chemotaxis. In addition, the deficiency of Bam32, as well as the inhibition of Rap1 activation, increased the levels of CXCL2-induced Akt1/2 phosphorylation at Thr308/309 in neutrophils. The inhibition of Akt by SH-5 attenuated CXCL2-induced adhesion and emigration in Bam32^−/−^ mice. Together, our results reveal that Bam32 has a suppressive role in chemokine-induced neutrophil chemotaxis by regulating Rap1 activation and that this role of Bam32 in chemokine-induced neutrophil recruitment relies on the activation of PI3K effector Akt.

## 1. Introduction

Neutrophil recruitment is a dynamic multi-step process that is regulated by a variety of molecules and signaling cascades elicited by the activation of Gi-coupled chemoattractant and chemokine receptors during acute inflammation [1]. Much of the research evidence in vivo and in vitro has indicated that one PI3K isoform, PI3Kγ, plays a critical role in the initial signal transduction downstream of the chemokine receptors [2,3,4]. Upon stimulation by various chemoattractants, PI3Kγ increases the localized accumulation of membrane-captive phospholipid PI(3,4,5)P3, which directs neutrophil movement and remodeling through binding recruited PI3K adaptors and effectors at the leading edge underneath the cell membrane [4]. The affinity of this interaction may vary: some signaling proteins such as Grp1 and Btk preferentially bind PI(3,4,5)P3 [5,6], some signaling proteins such as TAPP1/2 preferentially bind PI(3,4)P2 [7], and some others such as Akt bind both [8]. The functions of PI3K effector Akt were well-studied previously, but the roles for the most adaptors remain unclear. 

The B cell adaptor molecule of 32 kDa (Bam32), also known as dual adaptor for phosphotyrosine and 3-phosphoinositides 1 (DAPP1), is one of the PI3K adaptors that interacts with PI(3,4,5)P3 and its breakdown product PI(3,4)P2, regulating downstream cell growth, proliferation, and differentiation [9]. Bam32 is widely expressed in leukocytes including B lymphocytes, T lymphocytes, mast cells, and dendritic cells (DCs) [10], in which the roles of Bam32 have been uncovered in recent years. In mouse B lymphocytes, Bam32 induces the cytoskeletal rearrangement, actin remodeling, membrane ruffling, and lamellipodium formation by regulating Rac1 activation [11]. It also increases B cell adhesion and spreading on integrin ligands by activating Rho family GTPases [12]. Bam32 in mast cells inhibits FcƐRI-induced calcium flux and granule release by activating Lyn and SHIP negative signaling pathways [13]. Furthermore, Bam32 in DC is located at the DC-T cell contact sites and binds to galectin-1 to suppress the FOXP3^+^-induced proliferation of T cells [14]. Bam32 is expressed at similar levels in mouse neutrophils as in B cells [15]. However, the role of Bam32 in chemokine-induced neutrophil recruitment remains completely un-defined. 

In this study, we investigated the role of Bam32 in mouse neutrophil migration and chemotaxis, both in vivo and in vitro. By using real-time intravital microscopy and time-lapsed movie analysis, we examined chemokine CXCL2-induced neutrophil transmigration and chemotaxis in the cremaster muscle and neutrophil recruitment in the peritoneum of Bam32^−/−^ mice. Moreover, we determined CXCL2-induced chemotaxis in isolated Bam32^−/−^ neutrophils. By analyzing the activation of small GTPases Rac1, Rac2, and Rap1; the phosphorylation of Akt in CXCL2-stimulated neutrophils; and the effects of corresponding inhibitors in both in vivo and in vitro models, we unraveled the underlying Bam32-dependent mechanisms involved in CXCL2-induced transmigration and chemotaxis of neutrophils in mice. 

## 2. Results

### 2.1. Deficiency of Bam32 Increases CXCL2-Induced Neutrophil Transmigration and Chemotaxis in Mouse Cremaster Muscle

**Figure 1:**
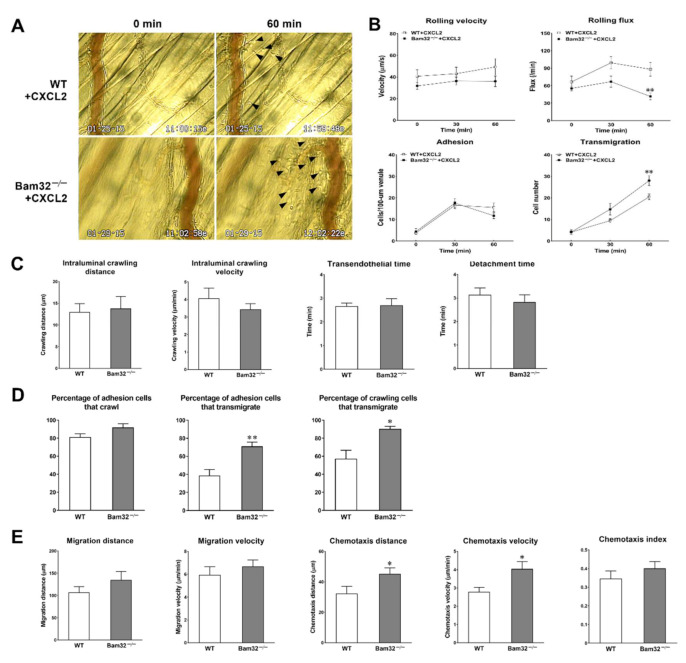
Effect of Bam32 deficiency on CXCL2-induced neutrophil recruitment in the mouse cremaster muscle. (**A**) Representative micrographs from intravital video microscopy showing a postcapillary venule and the surrounding tissue of the cremaster muscle with transmigrated neutrophils (arrow head) before (0 min) and at 60 min following the placement of CXCL2-containing gel (out of the view, 350-µm distant from the venule) in wild-type (WT) and Bam32^−/−^ mice (magnification: 100×). (**B**) Intravascular leukocyte rolling velocity, rolling flux, and numbers of adherent neutrophils (cells/100-µm venule) and emigrated neutrophils (cells/235 × 208 µm^2^ field) before (0 min) and 30 and 60 min after the placement of CXCL2-containing gel on the cremaster muscle of WT and Bam32^−/−^ mice. (**C**) The crawling distance (µm), crawling velocity (µm/min), transendothelial time (min), and detachment time (min) during 60-min video recording following the placement of CXCL2-containing gel in WT and Bam32^−/−^ mice. (**D**) The percentage of adherent cells that crawled on the luminal surface of the endothelium, the percentage of adhesion cells that transmigrated across the endothelium, and the percentage of crawling cells that transmigrated across the endothelium during 60-min video recording following the placement of CXCL2-containing gel in WT and Bam32^−/−^ mice. (**E**) The migration distance (µm), chemotaxis distance (µm), migration velocity (µm/min), chemotaxis velocity (µm/min), and chemotaxis index of neutrophil chemotaxis in extravascular tissue during 60 min following the placement of CXCL2-containing gel on the cremaster muscle of WT and Bam32^−/−^ mice (averaged from >80 cells). (**B**−**E**): mean ± SEM of 8 mice per group. * and ** indicate significant differences (*p* < 0.05 and *p* < 0.01, respectively) from WT mice.

First, we explored the role of Bam32 in CXCL2-induced neutrophil recruitment in mouse cremasteric post-capillary venules by using intravital microscopy. As shown in Figure 1A,B, the deficiency of Bam32 significantly decreased intravascular leukocyte rolling flux and increased neutrophil transmigration after 60 min of treatment with CXCL2 without changing rolling velocity or neutrophil adhesion. By analyzing the time-lapsed movies, we further determined which recruitment steps of the entire recruitment process were enhanced by the deficiency of Bam32. Figure 1C shows that the deficiency of Bam32 failed to change the neutrophil intraluminal crawling distance (the average total distance (µm) traversed by crawling neutrophils on the luminal surface of endothelium), intraluminal crawling velocity (the average velocity (µm/min) of crawling neutrophils on the luminal surface of endothelium), the transmigration time (the duration (min) of neutrophil transmigration across the endothelium), or detachment time (the average time (min) of transmigrated neutrophils detached from the venule) of transmigrated neutrophils. By quantifying the numbers of total adherent, crawling, and transmigrated cells, we found that the percentages of adhesion and crawling cells that transmigrated, but not the percentage of adherent cells that crawled, were significantly increased by Bam32 deficiency (Figure 1D). Moreover, we quantified the migration distance (the total distance (µm) traversed by an average migrating neutrophil in extravascular tissue), migration velocity (the velocity (µm/min) of neutrophil migration in extravascular tissue), chemotaxis distance (chemotactic distance (µm) in the direction to the source of CXCL2 but perpendicular to the venule traversed by a chemotactic neutrophil in extravascular tissue), chemotaxis velocity (the velocity (µm/min) neutrophil chemotaxis in the direction to the source of CXCL2), and chemotaxis index (the ratio of the chemotaxis distance divided by the migration distance). Figure 1E shows that, compared to wild-type (WT) neutrophils, the extravascular Bam32^−/−^ neutrophils demonstrated significantly increased chemotaxis distance and chemotaxis velocity, but similar migration distance, migration velocity, and chemotaxis index (the last of which represents the directionality of neutrophil movement) after transmigrating into cremaster muscle tissue. Together, these results indicated that the deficiency of Bam32 enhances CXCL2-induced neutrophil transmigration and chemotaxis in the mouse cremaster muscle. 

### 2.2. Deficiency of Bam32 Increases CXCL2-Induced Neutrophil Emigration in Mouse Peritoneum

**Figure 2:**
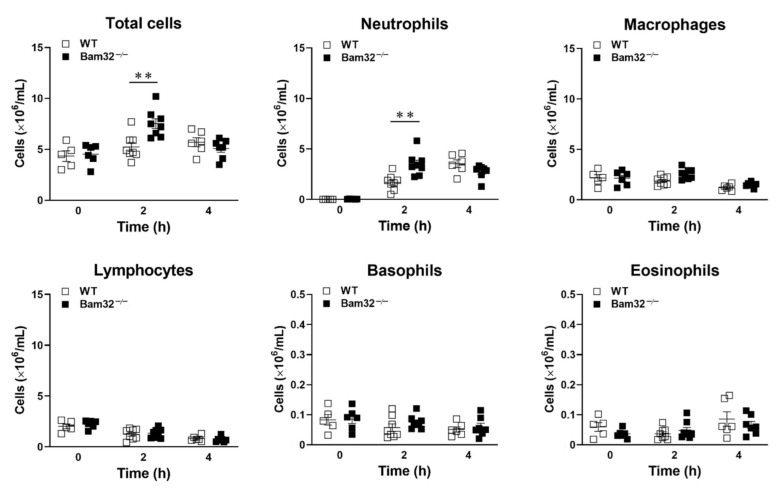
Effect of Bam32 deficiency on CXCL2-induced neutrophil emigration in the mouse peritoneum. The numbers of total emigrated leukocytes, neutrophils, macrophages, lymphocytes, basophils, and eosinophils were counted in peritoneal lavage fluid before (0 h) and 2 or 4 h after the intraperitoneal injection of CXCL2 (0.5 µg/25 g body weight). Mean ± SEM of 5−8 mice per group. ** indicates significant differences (*p* < 0.01) between WT and Bam32^−/−^ mouse strains.

Because neutrophils in different organs respond differently to even the same stimulus during inflammation [16], we explored whether the deficiency of Bam32 enhances CXCL2-induced neutrophil recruitment in the mouse peritoneum. As shown in Figure 2, the deficiency of Bam32 significantly increased the numbers of total emigrated leukocytes and neutrophils after a 2-h treatment with CXCL2. Interestingly, after a 4-h treatment with CXCL2, the significant differences in the numbers of total emigrated leukocytes and neutrophils between Bam32^−/−^ and WT mice disappeared. These results indicated that Bam32 may only function in CXCL2-induced neutrophil recruitment at an early time point (2 h), implicating a rapid but transient role of Bam32 in mouse peritonitis. 

### 2.3. Deficiency of Bam32 Increases CXCL2-Induced Neutrophil Chemotaxis In Vitro

**Figure 3:**
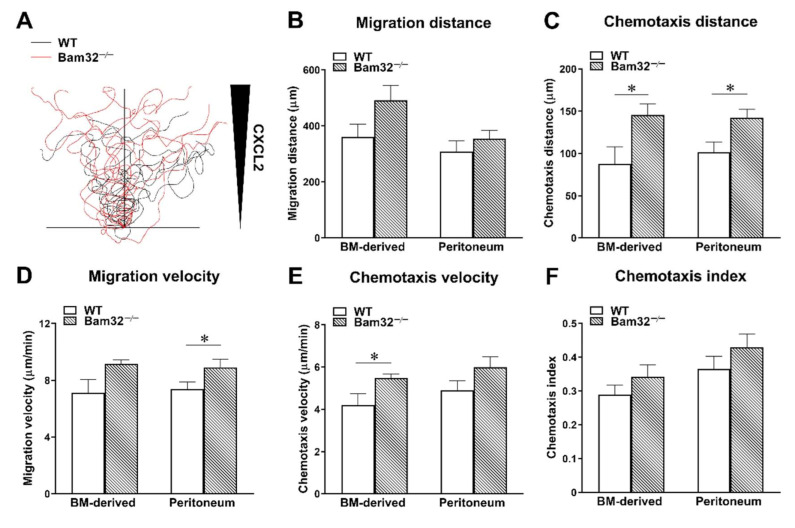
Effect of Bam32 deficiency on CXCL2-induced chemotaxis of bone marrow-derived neutrophils and pre-stimulated peritoneum neutrophils in vitro. Spider plots (**A**) display the chemotactic paths of representative 10 individual neutrophils from one mouse of each strain. The migration distance in µm (**B**), chemotaxis distance in µm (**C**), migration velocity in µm/min (**D**), chemotaxis velocity in µm/min (**E**), and chemotaxis index of isolated neutrophils for 60 min in 2D chemotaxis µ-slide were quantified and averaged from >50 cells in each group (**F**). Mean ± SEM of 4 mice per group. * indicates significant difference (*p* < 0.05) between WT and Bam32^−/−^ mouse strains. BM, bone marrow and chemotaxis index of isolated neutrophils for 60 min in 2D chemotaxis µ-slide were quantified and averaged from >50 cells in each group (**F**).

The neutrophil–endothelium interaction was reported to play a dominant role in neutrophil extravasation during acute inflammation by many previous studies [17]. To investigate the role of Bam32 in neutrophils without involving the endothelium, we determined CXCL2-induced migration distance (the total distance (µm) migrated by an average chemotactic neutrophil in the ibidi^®^ chamber), migration velocity (the velocity (µm/min) of neutrophil migration in the ibidi^®^ chamber), chemotaxis distance (the chemotactic distance (µm) in the direction to the source of CXCL2 migrated by a chemotactic neutrophil in the ibidi^®^ chamber), chemotaxis velocity (the velocity (µm/min) of neutrophil chemotaxis in the direction to the source of CXCL2 in the ibidi^®^ chamber), and chemotaxis index (the ratio of dividing the chemotaxis distance by the migration distance) of bone marrow-derived primary neutrophils and of oyster glycogen, pre-stimulated, peritoneal neutrophils in vitro. Each individual migrating neutrophil was tracked, and the trace was plotted (Figure 3A). As shown in Figure 3, in bone marrow-derived neutrophils, the deficiency of Bam32 significantly increased neutrophil chemotaxis distance (Figure 3C) and chemotaxis velocity (Figure 3E) in vitro, an observation that was consistent with our results obtained in the CXCL2-treated mouse cremaster muscle. Interestingly, in pre-stimulated, peritoneum neutrophils, the deficiency of Bam32 increased chemotaxis distance (Figure 3C) and migration velocity (Figure 3D) but not chemotaxis velocity (Figure 3E). This minor discrepancy might have been due to the activation of Toll-like receptors by oyster glycogen during neutrophil stimulation in the peritoneum in addition to the activation of receptors for CXCL2 [18]. Together, these results provide further evidence that the deficiency of Bam32 enhances CXCL2-induced neutrophil migration and chemotaxis in vitro. 

### 2.4. Deficiency of Bam32 Downregulates the Activation of Small GTPase Rap1

**Figure 4:**
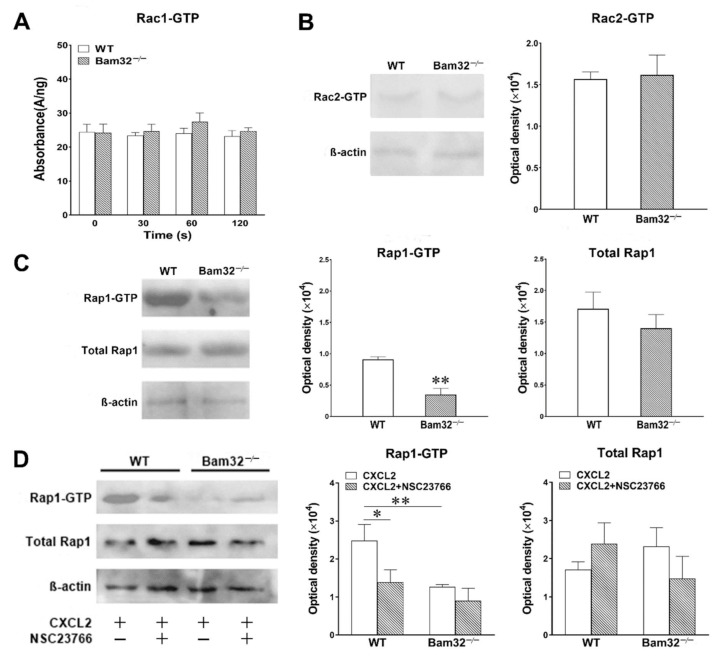
Effect of Bam32 deficiency on CXCL2-induced Rac1, Rac2, and Rap1 activation in neutrophils. (**A**) Levels of Rac1-GTP in bone marrow-derived neutrophils from WT and Bam32^−/−^ mice with saline (0 s) and after the stimulation of neutrophils with CXCL2 (0.1 µM) for 30, 60, and 120 s. (**B**,**C**) Representative original Western blots and the levels of Rac2-GTP (**B**) and Rap1-GTP (**C**) determined in bone marrow-derived neutrophils from WT and Bam32^−/−^ mice at 2 min after the stimulation of neutrophils with CXCL2 (0.1 µM). (**D**) Representative original Western blots and the levels of Rap1-GTP and total Rap1 determined in bone marrow-derived neutrophils from WT and Bam32^−/−^ mice at 2 min after the stimulation of neutrophils with CXCL2 (0.1 µM) and with pre-treatment with DMSO (vehicle) or Rac1 inhibitor NSC23766 (50 µM) for 30 min and throughout the 2-min CXCL2 stimulation. (**A**−**D**): mean ± SEM of 3 samples per group. Each sample was individually collected and pooled from two mice of the same strain. The Rac1 G-LISA assay was performed twice in the 96-well plate. * and ** indicate significant differences (*p* < 0.05 and *p* < 0.01, respectively) from WT mice (**C**,**D**) or from WT mice without NSC23766.

In view of the modulatory effects of Bam32 on the small GTPases required for cytoskeletal rearrangement after the activation of CXC chemokine receptor in B cells [11], we investigated CXCL2-induced activation of small GTPases Rac1, Rac2, and Rap1 by measuring GTP-bound Rac1, Rac2, and Rap1 in bone marrow-derived WT and Bam32^−/−^ neutrophils. As shown in Figure 4A,B, the deficiency of Bam32 failed to change the levels of activated GTP-bound Rac1 and Rac2 in neutrophils activated by CXCL2. Figure 4C shows that the deficiency of Bam32 resulted in the suppression of GTP-bound Rap1 activation with CXCL2 stimulation but had no apparent effect on changing the total Rap1 expression. Given that Rac1 activation is required for Rap1 activation in neutrophils [19], we then explored whether the inhibition of Rac1 activation reduced the activation of Rap1 in CXCL2-stimulated neutrophils by applying Rac1 inhibitor NSC23766. As shown in Figure 4D, the inhibition of Rac1 activation suppressed Rap1 activation in WT mice without changing the expression of total Rap1. Our data indicated that the presence of Bam32 and the activation of Rac1 are both required for Rap1 activation in mouse neutrophils. 

### 2.5. Inhibition of Rap1 Increases Neutrophil Chemotaxis In Vitro

**Figure 5:**
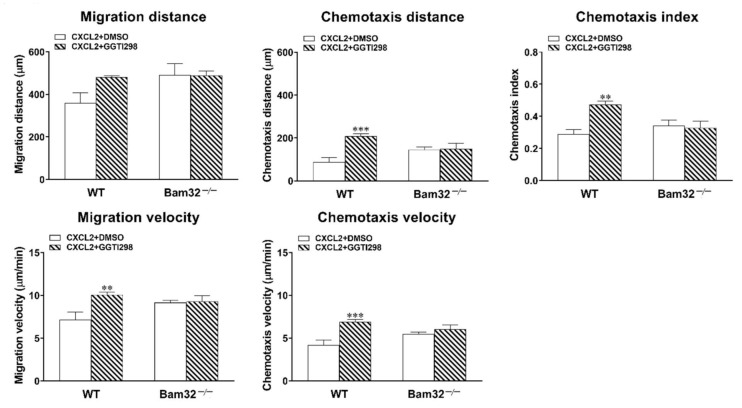
Effects of Rap1 inhibition on CXCL2-induced neutrophil chemotaxis in vitro. Data shown are the migration distance (µm), chemotaxis distance (µm), chemotaxis index, migration velocity (µm/min), and chemotaxis velocity (µm/min) for 60 min of neutrophils treated with DMSO (vehicle) or GGTI298 (10 µM)—a geranylgeranyltransferase I inhibitor for blocking the processing of geranylgeranylated Rap1—in 2D chemotaxis µ-slide (averaged from >50 cells). Mean ± SEM of 4 mice per group. ** and *** indicate significant differences (*p* < 0.01 and *p* < 0.001, respectively) from the WT neutrophils with DMSO only.

To investigate whether the modulation of Rap1 activation changes CXCL2-induced neutrophil chemotaxis, we incubated fresh bone marrow-derived neutrophils with GGTI298 (geranylgeranyltransferase I inhibitor; 10 µM) for 30 min prior to and throughout 1-h CXCL2 stimulation in vitro. The application of GGTI298 strongly inhibited the processing of geranylgeranylated Rap1 with little effect on other small GTPases in the Ras subfamily [20,21]. As depicted in Figure 5, the inhibition of Rap1 activation failed to enhance the chemotaxis of CXCL2-treated Bam32^−/−^ neutrophils, but it significantly increased the chemotaxis distance, chemotaxis velocity, migration velocity, and chemotaxis index of CXCL2-treated WT neutrophils. Thus, our results suggested that modulating the activation of Rap1 regulates downstream CXCL2-induced neutrophil chemotaxis in vitro. 

### 2.6. Deficiency of Bam32 Increases CXCL2-Induced Akt Phosphorylation at Site Thr308/309

**Figure 6:**
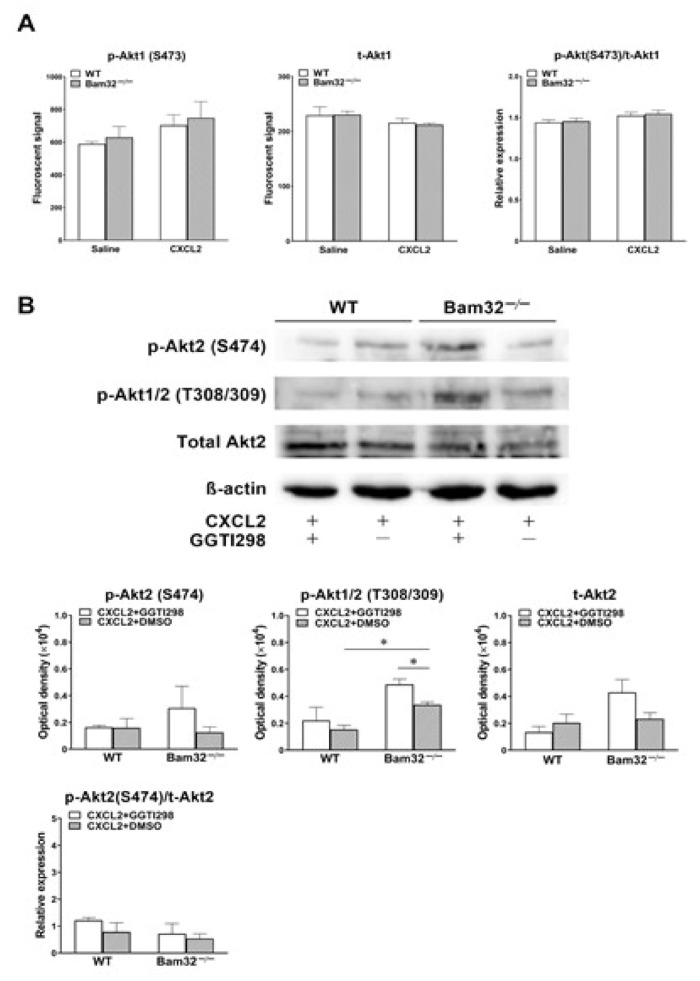
Effect of Bam32 deficiency on CXCL2-induced phosphorylation of Akt in neutrophils. (**A**) The levels of phospho-Akt1 (p-Akt1) at Ser473 and total Akt1 (t-Akt1), as well as the relative expression determined in bone marrow-derived WT and Bam32^−/−^ neutrophils treated for 5 min with saline or CXCL2 (0.1 µM). (**B**) Representative original Western blots and the levels of phospho-Akt2 (p-Akt2) at S474, phospho-Akt1/2 (p-Akt1/2) at Thr308/309, total Akt2 (t-Akt2), and the relative expression of phospho-Akt2 (p-Akt2) at S474 over total Akt2 (t-Akt2) determined in bone marrow-derived WT and Bam32^−/−^ neutrophils stimulated for 5 min with CXCL2 (0.1 µM) and with the treatment of DMSO (vehicle) or GGTI298 (10 µM) for 30 min prior to and throughout the 5-min CXCL2 stimulation. (**A**,**B**): mean ± SEM of 3 samples per group. Each sample was individually collected and pooled from two mice of the same strain. * indicates significant difference between the two groups (*p* < 0.05).

Because the activation of Rap1 was revealed to suppress the phosphorylation of Akt, which is the key modulator downstream of PI3K to regulate chemoattractant-induced recruitment in mouse neutrophils [22], we explored whether the Bam32 deficiency that impaired Rap1 activation changed CXCL2-induced phosphorylation of Akt. As shown in Figure 6A,B, the deficiency of Bam32 had no effect on the levels of total Akt1 and Akt2 or CXCL2-induced phosphorylation of Akt at Ser473 and Ser474, but it enhanced CXCL2-induced phosphorylation of Akt1/2 at Thr308/309 in neutrophils. However, the inhibition of Rap1 by GGTI298 increased the phosphorylation of Akt1/2 at Thy308/309 in Bam32^−/−^ neutrophils treated with CXCL2. Thus, our results suggested that Bam32-dependent Rap1 activation suppresses Akt phosphorylation in CXCL2-stimulated neutrophils. Moreover, Bam32-independent Rap1 activation also has a suppressive effect but may be inferior to Bam32-dependent Rap1 activation on the phosphorylation of Akt at Thr308/309. 

### 2.7. Inhibition of Akt Suppresses the Enhanced Neutrophil Transmigration in CXLC2-Treated Bam32^−/−^ Mice

**Figure 7:**
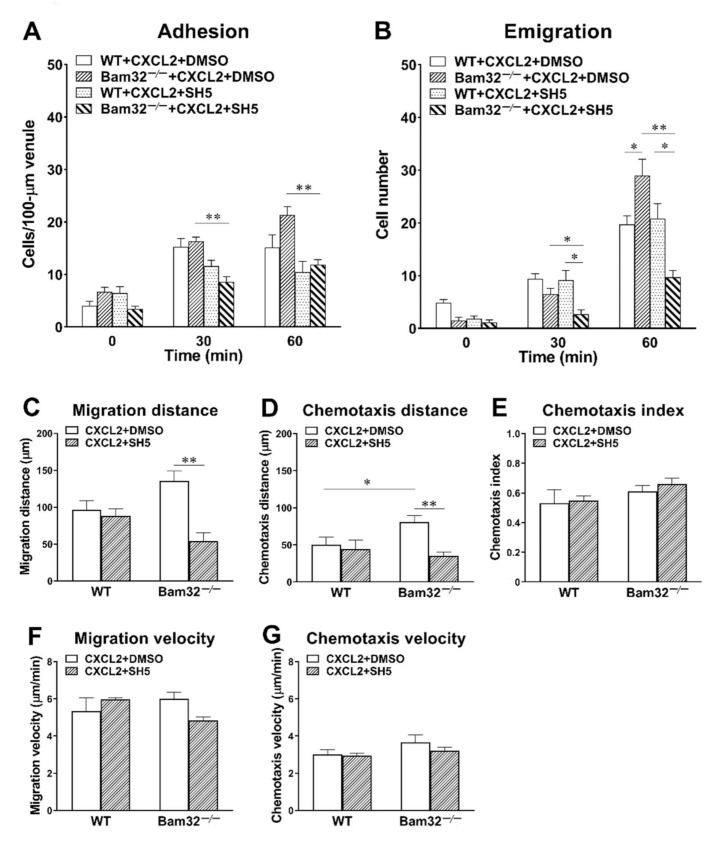
Effect of Akt inhibition on CXCL2-induced neutrophil recruitment in the mouse cremaster muscle. (**A**) The number of adherent neutrophils (cells/100-µm venule) and (**B**) the number of emigrated neutrophils (cells/235 × 208 µm^2^ field) before (0 min) and at 30 and 60 min following the placement and holding of CXCL2-containing gel on the cremaster muscle of WT and Bam32^−/−^ mice superfused with DMSO (vehicle) or the specific pan-Akt inhibitor SH-5 (1 µM) for 30 min prior to and throughout the 60-min CXCL2 stimulation. (**C**−**G**) The migration distance, chemotaxis distance, chemotaxis index, migration velocity, and chemotaxis velocity of neutrophils in extravascular tissue during 60 min following the placement of CXCL2-containing gel on the cremaster muscle of WT and Bam32^−/−^ mice (averaged from >50 cells) superfused with DMSO (vehicle) or SH-5 (1 µM) for 30 min prior to and throughout the 60-min CXCL2 stimulation. (**A**−**G**): mean ± SEM of 4 mice per group. * and ** indicate significant differences (*p* < 0.05 and *p* < 0.01, respectively) between treatments with DMSO (vehicle) and SH-5 (**C**−**G**) in CXCL2-treated Bam32^−/−^ mice.

Here, we sought to elucidate whether the inhibition of Akt attenuated CXCL2-induced enhancement in neutrophil recruitment in the mouse cremaster muscle, which resulted from the deficiency of Bam32. SH-5 (specific pan-Akt inhibitor, 1 µM) was superfused on the cremaster muscle for 30 min prior to and throughout 1-h stimulation with CXCL2. As shown in Figure 7A,B, SH-5 failed to change the numbers of adherent and transmigrated neutrophils in WT mice, but it significantly decreased the numbers of adherent and transmigrated neutrophils in Bam32^−/−^ mice at both 30 and 60 min. Interestingly, after the application of SH-5, the numbers of adherent and transmigrated neutrophils in Bam32^−/−^ were significantly lower than those in WT mice, suggesting that the Bam32-dependent enhancement in CXCL2-induced neutrophil recruitment in vivo relies on the activation of Akt.

By further analyzing the time-lapsed movie, we found that the inhibition of Akt with SH-5 significantly decreased migration distance (Figure 7C) and chemotaxis distance (Figure 7D) without changing the directionality of neutrophil chemotaxis (chemotaxis index, Figure 7E), migration velocity (Figure 7F), or chemotaxis velocity (Figure 7G) in Bam32^−/−^ mice. These data indicated that the activation of Akt, which is suppressed by Bam32^−/−^-dependent Rap1 activation, is required in CXCL2-induced neutrophil recruitment in mice.

## 3. Discussion

Mounting research evidence has demonstrated that the activation of PI3K and its downstream adaptors and effectors is implicated in orchestrating neutrophil recruitment during acute inflammation [23,24]. Our study is the first to unveil a hitherto unknown suppressive role of Bam32, a PI3K downstream adaptor originally discovered in B cells, in neutrophil transmigration and chemotaxis induced by a CXC chemokine in vivo and in vitro. We showed that the deficiency of Bam32 enhanced CXCL2-induced neutrophil transmigration in the mouse cremaster muscle and peritoneum at early time points, and we revealed that the deficiency of Bam32 in neutrophils increased CXCL2-induced neutrophil chemotaxis distance and velocity without involving endothelial cells in vitro. The underlying mechanism of the suppressive role of Bam32 in neutrophil chemotaxis in vitro was the modulation of Rap1 activation, which was evidenced by the measurements of neutrophil Rap1 activation, migration, and chemotaxis after the application of GGTI298 (a geranylgeranyltransferase I inhibitor for blocking the processing of geranylgeranylated Rap1) to bone marrow-derived neutrophils from both mouse strains. However, this Bam32/Rap1 pathway was found to regulate neutrophil chemotaxis in vitro independent of the activation of Akt, which was different from the in vivo mouse cremaster muscle model where the PI3K effector Akt was found to play a pivotal role in regulating CXCL2-induced, Bam32-dependent neutrophil recruitment.

A supportive role of Bam32 was reported in modulating BCR-induced, F-actin-mediated cytoskeletal remodeling in B lymphocytes [11]. However, our observation points to a suppressive role of Bam32 in regulating chemokine-induced neutrophil recruitment, because the deficiency of Bam32 substantially increased CXCL2-induced neutrophil transmigration in the mouse cremaster muscle. This augment in neutrophil transmigration may have been due to the increased initiation of transmigration, as evidenced by the increased percentages of adhesion cells that transmigrated and crawling cells that transmigrated through a Bam32-dependent mechanism. Though neutrophil adhesion is regarded as one of the important prerequisites for neutrophil transmigration [1,25,26], our data showed no difference in neutrophil adhesion between WT and Bam32^−/−^ mice. It is reasonable to consider that the deficiency of Bam32 only resulted in a 50%, albeit significant, decline in leukocyte rolling flux at 60 min, which was insufficient to significantly increase neutrophil adhesion at 60 min or earlier time points. Therefore, our results exclude the possibility that the Bam32 deficiency-involved increase in neutrophil transmigration was due to increased rolling or adhesion. In extravascular tissue, the deficiency of Bam32 increased neutrophil chemotaxis distance and velocity without changing the migration distance, migration velocity, or chemotaxis index. This suggests that Bam32 may be involved in neutrophil chemotactic movement without influencing other neutrophil activities such as chemotactic directionality in the mouse cremaster muscle. Interestingly, we found that the deficiency of Bam32 had no effects on TNFα-induced neutrophil adhesion and transmigration (Figure S1) but decreased WKYMVm-induced neutrophil adhesion and transmigration (Figure S2), suggesting that the role of Bam32 in regulating neutrophil recruitment may be stimulus-specific.

Though the classical paradigm of neutrophil recruitment has largely been established in the mouse cremaster muscle and mesentery by the use of intravital microscopy [27], recruitment mechanisms may deviate in different organs. This deviation is due to organ-specific vasculature, perivascular cells, and organ-specific tissue resident cells [28,29]. In our study, the suppressive role of Bam32 in chemokine-induced mouse neutrophil recruitment was verified in CXCL2-induced neutrophil emigration in the mouse peritoneum. The deficiency of Bam32 increased the emigrated neutrophil number as early as 2 h after intraperitoneal injection of CXCL2, a result that was in line with our observation of neutrophil recruitment within 1 h of CXCL2 treatment in the mouse cremaster muscle. Intriguingly, in CXCL2-induced mouse peritonitis, the difference that resulted from the deficiency of Bam32 in the number of emigrated neutrophils was diminished at 4 h after intraperitoneal injection of CXCL2, implying that the Bam32-dependent regulation of neutrophil emigration may only be responsive to CXCL2 at early time points. However, our study cannot exclude the possibility that longer treatment with CXCL2 may still trigger more emigrated cells but reduce the “lavagibility” of those emigrated neutrophils by strengthening their binding to the peritoneum, since the continuous stimulation with CXCL2 may increase the expression of adhesion molecules on the surface of emigrated neutrophils and other peritoneal cells.

The neutrophil-endothelial adhesive interaction is a sophisticated process that regulates subsequent neutrophil extravasation from the microvasculature [17,26,30,31]. Neutrophil firm adhesion mediated by β_2_-integrin is the fundamental step that triggers the intrinsic signaling for neutrophil transmigration [32], which includes two distinct transcellular and paracellular transmigration pathways [33]. Here, we scrutinized the role of Bam32 in neutrophils by determining the CXCL2-induced extravascular chemotaxis of isolated neutrophils from the two mouse strains in vitro. Our data showed that, consistent with our recruitment results in vivo, the deficiency of Bam32 enhanced CXCL2-induced neutrophil chemotaxis in vitro.

Rap1, a member of Ras family of small GTPases, has a regulatory role in neutrophil recruitment. Rap1a is required for E-selectin-dependent slow rolling in leukocytes after the activation of the CalDAG-GEFI and p38 MAPK signal pathways [34]. Moreover, Rap1b suppresses neutrophil transendothelial migration and chemotaxis by inhibiting Akt in the PI3K signaling pathway [35]. Our results strongly indicated that Bam32 is required for Rap1 activation, which suppresses CXCL2-induced neutrophil chemotaxis. Interestingly, as shown in our study and elsewhere [19], Bam32 is not the only prerequisite for Rap1 activation. The activation of Rac1, a Rho family small GTPase that is regulated by Bam32 in B lymphocytes through a tyrosine phosphorylation-dependent mechanism [11], is equally important for CXCL2-induced Rap1 activation in mouse neutrophils. Nevertheless, the mechanisms of how Bam32 modulates the activation of Rap1 warrant future studies.

The integrin α_M_β_2_-dependent Akt signaling in neutrophil recruitment is regulated by Rap1b, as Rap1b deficiency enhances Akt phosphorylation by increasing α_M_β_2_ cross-linking [35]. Akt has three different isoforms: Akt1, Akt2 and Akt3; Akt1 and Akt2 are involved in leukocyte migration and Akt3 controls the growth and development of mice and humans [36,37,38]. In previous studies, Akt1 in endothelial cells was found to increase B-cell migration by activating STAT5 [39] and to enhance neutrophil migration by elevating microvascular permeability [40]. By contrast, Akt2 performs its role in neutrophils by controlling neutrophil degranulation and migration. This identified mechanism challenges the existing paradigm that all Akt proteins have similar functions in neutrophil recruitment [22]. The in vitro model of our study showed that the deficiency of Bam32 increased Akt phosphorylation, a result consistent with the previous study [35]. However, the pharmacological inhibition of Rap1 increased chemotaxis but not Akt phosphorylation in WT neutrophils, suggesting that the mechanism of enhanced chemotaxis in vitro is Rap1-dependent but Akt-independent. On the contrary, the Bam32-dependent suppressive role in CXCL2-induced neutrophil recruitment in vivo is Akt-dependent, which was evidenced by the suppression of neutrophil adhesion and emigration in Bam32^−/−^ mice but not in WT mice after the application of the pan-Akt inhibitor SH-5. In this regard, our study revealed similar suppressive roles of Bam32 in neutrophil recruitment and chemotaxis through possibly different mechanisms in vitro and in vivo.

It is noteworthy that the GGTI298 used in our study is not a specific Rap1 inhibitor but a geranylgeranyl transferase I inhibitor that blocks the processing of the Ras family and prevents the attachment of small GTPases to the cell membrane. Rap1 is one of the substrates downstream of geranylgeranyl transferase I, and it can therefore be blocked by GGTI298. In many previous studies [21,41,42], GGTI298 (10 µM) could be used, albeit not entirely specifically, to block the activation of Rap1, with very little effects on the processing of other endogenous small GTPases in the Ras family [21,41]. However, we cannot completely rule out the possibility that Bam32 can regulate neutrophil chemotaxis in vitro through the activation of molecules other than Rap1.

## 4. Materials and Methods

### 4.1. Animals

Bam32^−/−^ mice were generated by Dr. Michel C. Nussenzweig [9], and then they were transferred to and maintained in the Lab Animal Services Unit at the University of Saskatchewan (Saskatoon, SK, Canada). Age-matched male WT C57BL/6N mice were purchased from Charles River Canada (Saint-Constant, QC, Canada). A total of 290, 6–12-week-old, age-matched male mice of these two strains were used in experiments. This study was carried out with the approved animal protocols (protocol number: 20070028) from the University Committee on Animal Care and Supply at the University of Saskatchewan and following the standards of Canadian Association of Animal Care. All efforts were made to reduce animal suffering, and all surgeries were performed under deep ketamine–xylazine anesthesia.

### 4.2. Intravital Microscopy

Mice were anesthetized after an intraperitoneal injection with a cocktail of ketamine (200 mg/kg, Rogar, Montreal, QC, Canada) and xylazine (10 mg/kg, Bayer, Toronto, ON, Canada). The mouse cremaster muscle was surgically exposed, as described previously [43] and was superfused with 37 °C-warmed bicarbonate-buffered saline (pH 7.4; containing, in mM, NaCl 133.9, KCl 4.7, MgSO_4_ 1.2, and NaHCO_3_ 20.0; all reagents were purchased from Thermo Fisher Scientific, Toronto, ON, Canada). An upright microscope (model Eclipse Ci-s, Nikon, Tokyo, Japan) with a 20× objective lens was used for bright-field intravital microscopy. A 2% agarose gel of 1-mm^3^ size containing the murine CXC chemokine CXCL2 (0.5 µM, R&D Systems, Minneapolis, MN, USA) was placed on the exposed cremaster muscle in a preselected area that was 350-µm distant from and parallel to the observed postcapillary venule. After been placed a 22 × 22 mm glass coverslip for holding the gel, the cremaster muscle was superfused with 37 °C-warmed bicarbonate-buffered saline at a very slow rate (≤10 µL/min) to allow for the formation of CXCL2 chemotactic gradient. All experiments showing neutrophil recruitment and hemodynamic changes in the selected cremasteric postcapillary venule (25–35 µm diameter) were recorded at real-time on a DVD video recorder upon (0 min) and after the addition of CXCL2-containing gel (for 60 min). During recording, all efforts were made to adjust and keep the microscopic images focused on the adhering, crawling, transmigrating, and chemotaxing neutrophils inside the venule and in extravascular cremaster muscle tissue. The rolling flux and velocity of leukocytes and the numbers of adherent and transmigrated neutrophils were determined in the cremasteric microvasculature during the offline playback analysis of the recorded real-time movies. A 512× time-lapsed movie was made from the 1-h real-time recording. The crawling distance, crawling velocity, transendothelial time, detachment time, migration distance, migration velocity, chemotaxis distance, chemotaxis velocity, and chemotaxis index were analyzed in the time-lapsed movie as described previously [44,45]. Where indicated, the specific Akt inhibitor SH-5 (1 µM, Santa Cruz Biotechnology, Inc., Mississauga, ON, Canada) was superfused on the cremaster muscle 30 min prior to and throughout the 1-h CXCL2 stimulation.

### 4.3. Cell Counting in CXCL2-Induced Acute Peritonitis

Emigrated neutrophils were harvested with an ice-cold lavage solution (pH 7.35; bovine serum albumin 2.5% *w*/*v*, in PBS) from the mouse peritoneum at 2 or 4 h after the intraperitoneal injection of CXCL2 (0.5 µg/25 g body weight), and then they were centrifuged at 300× *g* and 4 °C for 5 min. The cell pellet was resuspended in a 1-mL ice-cold cytospin solution (containing bovine serum albumin 10% *w*/*v* and NaN_3_ 3.1 mM in saline). The cell suspension (1 × 10^6^ mL, 150 µL) was loaded into the cytospin chamber. A film of cells on slides was prepared by cytocentrifugation, fixed and stained with Richard–Allan staining solutions (Thermo Fisher Scientific). The numbers of total emigrated leukocytes and neutrophils were morphologically quantified under a bright-field microscope (Magnification: ×200). 

### 4.4. Harvest of Emigrated Neutrophils from Peritoneum

Neutrophils were isolated from the peritoneum with the oyster glycogen-induced acute peritonitis of WT and Bam32^−/−^ mice. Oyster glycogen (10 mg/mL in sterile saline, Sigma-Aldrich, Oakville, ON, Canada) was injected into the mouse peritoneum for 2 or 4 h before emigrated neutrophils were harvested with an ice-cold lavage solution from the mouse peritoneum.

### 4.5. Harvest of Neutrophils from Mouse Bone Marrow

Neutrophils were freshly isolated from the bone marrow of WT and Bam32^−/−^ mice. The femur and tibia were dissected immediately after the mouse was euthanized by cervical dislocation. The marrow cavity was flushed with ice-cold calcium- and magnesium-free PBS. The bone marrow cells were enriched in flushing fluid, separated by three-density (72%, 64%, and 52%) Percoll (GE Healthcare, Baie d’Urfe, QC, Canada) gradient centrifugation at 1080× *g* for 30 min, and then washed in cold PBS as described previously [46]. This method yielded over 85% morphologically mature neutrophils [47].

### 4.6. Two-Dimensional (2D) Chemotaxis Assay In Vitro

Time-lapsed video microscopy was used to determine the distance, speed, and directionality of neutrophil chemotaxis in response to the CXCL2 chemokine gradient using a previously described method [48]. Primary neutrophils from either fresh bone marrow or emigrated neutrophils from the oyster glycogen-induced peritonitis were tested on uncoated ibiTreat Chemotaxis 2D µ-slide (ibidi^®^, Munich, Germany). Isolated neutrophils suspended in 37 °C Dulbecco’s Modified Eagle Medium (DMEM; ATCC, Manassas, VA, USA) containing 10% FBS were seeded into the narrow channel of the µ-slide strictly following the manufacturer’s user manual. After 30 min of incubation in a humidified incubator at 37 °C with 5% CO_2_, CXCL2 (18 µL and 6.25 µM) was loaded into one of the two opposing reservoirs, which were filled with a cell-free medium, to allow for the formation of the concentration gradient at the observation area. The chemotactic movements of the adherent neutrophils were recorded for 60 min at 37 °C using an ibidi^®^ heating and incubation chamber on an inverted microscope (Applied Precision, Woodbridge, ON, Canada) equipped with a CCD color video camera. The time-lapsed movie analysis of chemotaxis was performed using ImageJ software (National Institutes of Health, Bethesda, MD, USA), and at least 50 cells were tracked and analyzed for each group. Where indicated, the geranylgeranyltransferase I inhibitor GGTI298 (10 µM, Tocris, Oakville, ON, Canada) for Rap1 inhibition was incubated with neutrophils for 30 min prior to loading onto the µ-slide and remained throughout 1-h CXCL2 stimulation.

### 4.7. Rac1 Activation Assay

Fresh bone marrow-derived mouse neutrophils were prepared as described above and suspended in 37 °C serum-free DMEM. A colorimetric Rac1 G-LISA Activation Assay Kit (Cytoskeleton, Inc., Denver, CO) was used to quantitatively detect GTP-bound Rac1 in suspended neutrophils with saline (0 min) or CXCL2 (0.1 µM) for 30, 60, or 120 s. All steps of lysing neutrophils and extracting GTP-bound Rac1 were performed strictly following the instructions provided by the manufacturer. Protein concentrations were determined using the Precision Red Advanced Protein Assay that came with the kit. The fluorescent signal of each sample on 96-well plates was captured by a Fluoroskan Ascent Microplate Fluorometer (Thermo Fisher).

### 4.8. Bead-Based Rac2 Pull-Down Assay

Fresh bone marrow-derived mouse neutrophils were prepared as described above and suspended in 37 °C serum-free DMEM. GTP-bound Rac2 in neutrophils were determined using the Rac2 Activation Assay Kit (Abcam Canada, Toronto, ON, Canada). Briefly, neutrophils stimulated with CXCL2 (0.1 µM) were lysed in the assay/lysis buffer provided with the kit, followed by incubating with PAK1 PBD agarose beads at 4 °C for 1 h. The beads were washed with the assay/lysis buffer 3 times and then incubated with a 2× Laemmli sample buffer to elute GTP-bound Rac2. The elutes were electrophoresed in 12% SDS-PAGE, and the proteins were transferred to a nitrocellulose membrane before being incubated with rabbit anti-Rac2 Abs (1:1000 dilution; Abcam) for immunoblotting. The levels of β-actin in the total protein lysates were also blotted for the assessment of equal protein loading.

### 4.9. Resin-Based Rap1 Pull-Down Assay

Fresh bone marrow-derived mouse neutrophils were prepared as described above and suspended in 37 °C serum-free DMEM. After the 2-min stimulation of neutrophils with CXCL2 (0.1 µM) at 37 °C, total Rap1 and GTP-bound Rap1 in neutrophils were determined using the Active Rap1 Detection Kit (Cell Signalling Technology, New England Biolabs, Ltd., Whitby, ON, Canada) following the manufacturer’s protocol. Briefly, the total protein lysates were incubated with GST-bound RalGDS-RBD at 4 °C for 1 h. The resin was then washed and incubated with a 2× Laemmli sample buffer to elute GTP-bound Rap1. The elutes and total protein lysates were electrophoresed in 12% SDS-PAGE, and the proteins were transferred to a nitrocellulose membrane before being incubated with rabbit anti-Rap1 Abs (1:1000 dilution; Cell Signalling Technology) for immunoblotting. The levels of β-actin in the total protein lysates were also blotted for the assessment of equal protein loading.

### 4.10. Measurement of Akt Phosphorylation

The levels of total Akt1 and phospho-Akt1 (S473) were determined with Phospho (Ser473)/Total Akt Whole Cell Lysate Kit (Meso Scale Discovery, Rockville, MD, USA). The levels of total Akt2, phospho-Akt2 (S474), and phospho-Akt1/2 (T308/309) were determined with Western blotting. Briefly, neutrophil lysates in a RIPA buffer were mixed with a 4× Laemmli buffer (Volume ratio 2:1) at 95 °C for 5 min, resolved by 10% SDS-PAGE, transferred to a nitrocellulose membrane, and immunoblotted as described previously [47]. The nitrocellulose membrane was blocked with 5% BSA at room temperature for 1 h and then incubated at 4 °C overnight with rabbit anti-phospho-Akt2 (S474, 1:1000 dilution; Cell Signalling Technology) or rabbit anti-phospho-Akt1/2 (T308/309, 1:1000 dilution; Bioss, Woburn, MA, USA). After incubation with HRP-conjugated respective secondary Abs (1:4000 dilution; Enzo Life Sciences, Cedarlane, Burlington, ON, Canada), the protein binding was detected with Clarity ECL Substrates (Bio-Rad, Montreal, QC, Canada). The developed blots were washed with a stripping buffer (containing in mM, glycine 200, sodium dodecyl sulfate 3.47, and 1% Tween-20; pH 2.2) for 4 h and reprobed, respectively, with rabbit anti-Akt2 Abs (1:1000 dilution; Cell Signalling Technology) and mouse anti-β-actin Abs (1:2000 dilution; Invitrogen, Burlington, ON, Canada) antibodies, followed by incubation with corresponding secondary antibodies and Clarity ECL Substrates. Image Studio Lite (Version 5.2, LI-COR Biotechnology, Lincoln, NE, USA) was used for the densitometric quantification of the detected bands.

### 4.11. Statistic Analysis

Data are expressed as arithmetic means ± SEM from at least three independent experiments. Statistical analyses of the differences among groups were performed using two-sided Student *t* test or one-way ANOVA followed by the Holm–Sidak method, with *p* < 0.05 considered to be statistically significant. Analyses were conducted using GraphPad Prism 7 (GraphPad Software, La Jolla, CA, USA).

## 5. Conclusions

Our data showed an unprecedented suppressive role of Bam32 in regulating chemokine-induced mouse neutrophil transmigration and chemotaxis, both in vivo and in vitro. This Bam32-dependent suppression may be a functional breaking mechanism during neutrophil chemotaxis in vitro mediated by the small GTPase Rap1 and neutrophil recruitment in vivo mediated by the PI3K effector Akt. In this regard, our study provides a mechanistic insight into the role of the PI3K adaptor Bam32 in neutrophil recruitment and chemotaxis during chemokine-induced acute inflammation.

## Data Availability

The data presented in this study are available on request from the corresponding author.

## Supplementary Materials

The following are available online at https://www.mdpi.com/1422-0067/22/4/1825/s1.
