# Supplementary material for: Suppressive Role of Bam32/DAPP1 in Chemokine-Induced Neutrophil Recruitment
Source: Int J Mol Sci. 2021 Feb 12;22(4):1825. doi: 10.3390/ijms22041825 (PMC7918626; doi:10.3390/ijms22041825)
Supplement: Supplementary file 1 [file ijms-22-01825-s001.zip › Fig.S1 (210114).pdf]

Supplemental Figure S1

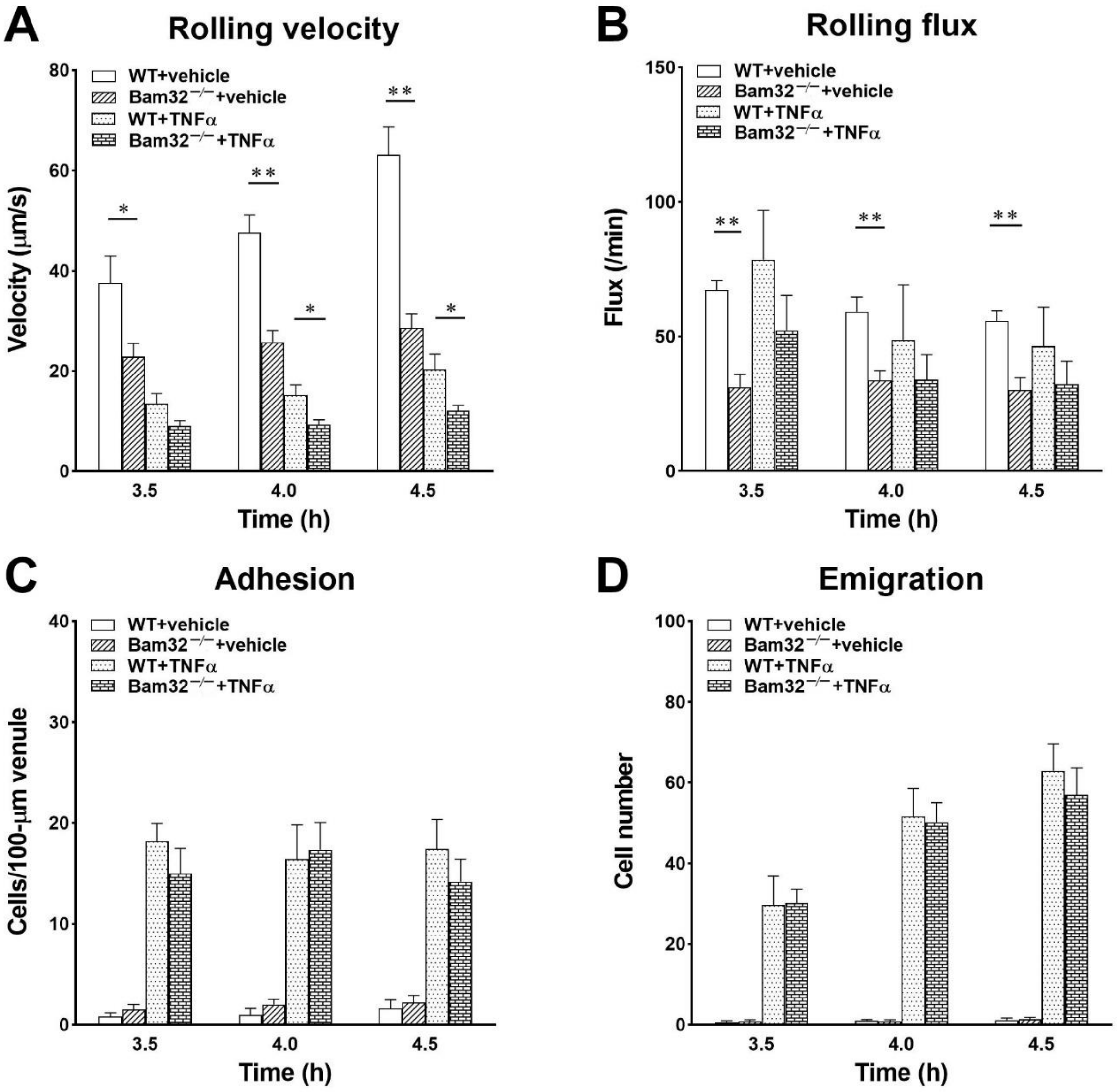

Fig.S1 Deficiency of Bam32 decreases TNFα-induced leukocyte rolling velocity and flux in mouse cremaster muscle. (A–D) Leukocyte rolling velocity, rolling flux, and the numbers of adherent neutrophils (cells/100-μm venule) and emigrated neutrophils (cells/235 × 416 μm^2 field) at 3.5 h, 4.0 h, and 4.5 h following intrascrotal injection of saline (vehicle) or recombinant murine TNFα (100 ng in 200 μl saline) in WT mice and $Bam32^{-/-}$ mice. A–D, mean ± SEM of 6 mice per group. */** indicate significant differences (*: $p<0.05$ and **: $p<0.01$) between WT and $Bam32^{-/-}$ mice.
