# Supplementary material for: Suppressive Role of Bam32/DAPP1 in Chemokine-Induced Neutrophil Recruitment
Source: Int J Mol Sci. 2021 Feb 12;22(4):1825. doi: 10.3390/ijms22041825 (PMC7918626; doi:10.3390/ijms22041825)
Supplement: Supplementary file 1 [file ijms-22-01825-s001.zip › Fig.S2 (210114).pdf]

Supplemental Figure S2

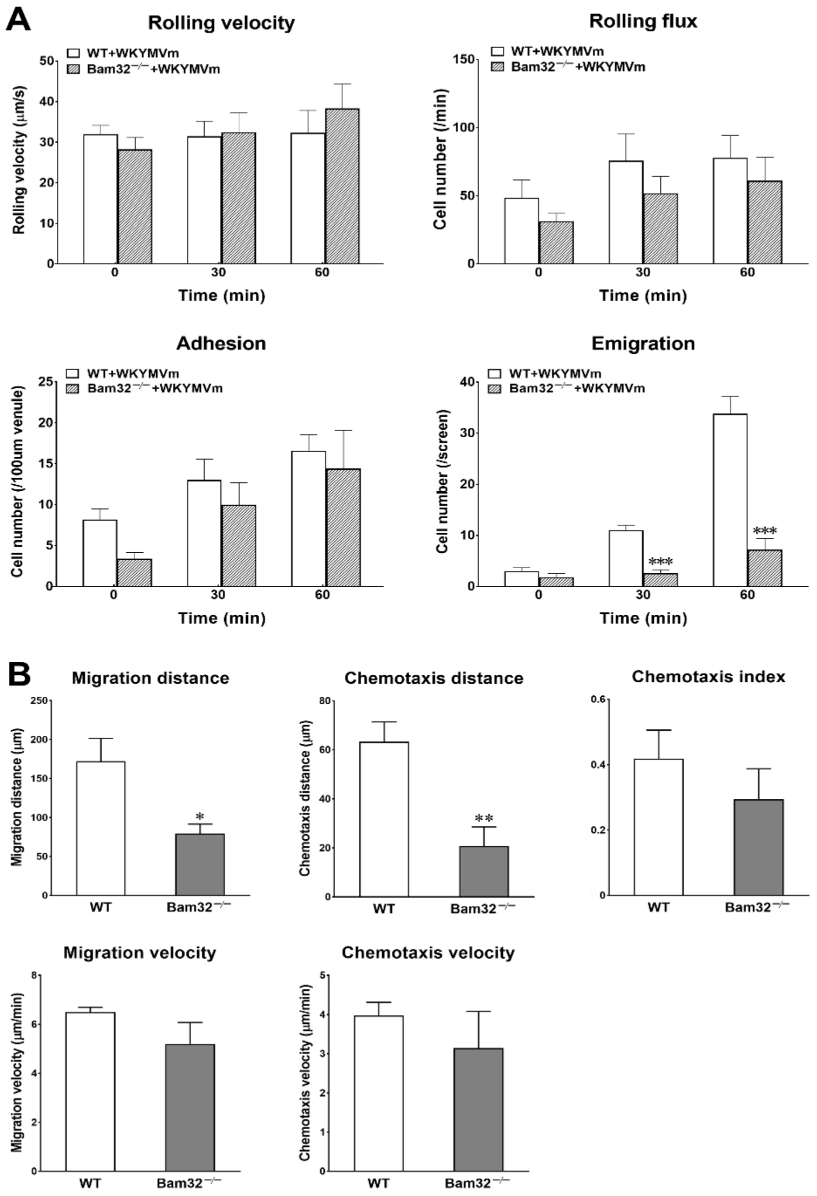

Fig.S2 Deficiency of Bam32 decreases WKYMVm-induced neutrophil recruitment and chemotaxis in mouse cremaster muscle. (A) Leukocyte rolling velocity, leukocyte rolling flux, neutrophil adhesion (cells/100-µm venule), and neutrophil emigration (cells/235 × 208 µm^2 field) at 0 min (before), 30 min and 60 min following the placement of WKYMVm-containing gel (0.1 mM of 1 mm^3) on cremaster muscle of WT and Bam32$^{-/-}$ mice. (B) The migration distance (µm), chemotaxis distance (µm), migration velocity (µm/min), chemotaxis velocity (µm/min), and chemotaxis index of neutrophils in extravascular tissue during 60 min following the placement of WKYMVm-containing gel on cremaster muscle of WT mice and Bam32$^{-/-}$ mice (averaged from >50 cells). A–B, mean ± SEM of 5 mice per group. */**/*** indicate significant differences (*: $p<0.05$, **: $p<0.01$, and ***: $p<0.001$, respectively) from WT mice.
